# Clinical efficacy and safety of platelet-rich plasma in arthroscopic full-thickness rotator cuff repair: A meta-analysis

**DOI:** 10.1371/journal.pone.0220392

**Published:** 2019-07-29

**Authors:** Chang Wang, Meng Xu, Wenlai Guo, Yaodong Wang, Shishun Zhao, Lei Zhong

**Affiliations:** 1 Department of Orthopedics, The Second Hospital of Jilin University, Changchun, China; 2 College of Mathematics, Jilin University, Changchun, China; 3 School of Science,China University of Mining & Technology, Beijing, China; University College London Hospitals NHS Foundation Trust, UNITED KINGDOM

## Abstract

**Background:**

Arthroscopic repair of rotator cuff tears, although commonly performed, carries the risk of retears. Therefore, bioremediation techniques such as platelet-rich plasma injections have been used as adjuvant therapies. The clinical efficacy of platelet-rich plasma in the arthroscopic repair of full-thickness rotator cuff injury is controversial. We performed a meta-analysis to evaluate the clinical effectiveness and safety of platelet-rich plasma and provide evidence-based medical recommendations for selecting the proper clinical treatment plan for full-thickness rotator cuff injuries.

**Methods:**

A search for the terms “platelet-rich plasma” and “rotator cuff” was performed in the PubMed, EMBASE, and Cochrane Library databases using a computer. After conducting quality evaluations and data extraction, RevMan 5.3 software was used to combine the effect sizes, and the GRADEpro Guideline Development Tool was used to rate the level of evidence from aspects of functional score, pain score and retear rate.

**Results:**

Eight randomized controlled trials involving 566 patients were included. The long-term retear rate(RR = 0.96, 95% CI [0.52, 1.78], P = .89), Constant score(RR = 0.96, 95% CI [0.52, 1.78], P = .89), and Visual Analog Scale score for pain (SMD = -0.28, 95% CI [-0.60, 0.04], P = .08), as well as both the long-term and short-term Disabilities of the Arm, Shoulder, and Hand scores(SMD = -0.13, 95% CI [-0.44, 0.18], P = .41;SMD = -0.02, 95% CI [-0.40, 0.36], P = .93), were not significantly different between the platelet-rich plasma and control groups. However, the short-term retear rate(RR = 0.29, 95% CI [0.13, 0.65], P = .003) and Visual Analog Scale score (SMD = -0.41, 95% CI [-0.62, -0.19], P = .0002) were significantly lower, while the short-term Constant score(SMD = 0.37, 95% CI [0.19, 0.55], P < .0001) and short-term and long-term University of California at Los Angeles activity scores (SMD = 0.38, 95% CI [0.16, 0.60], P = .0008;SMD = 0.85, 95% CI [0.48, 1.22], P < .00001) were significantly higher, in the platelet-rich plasma group than in the control group.

**Conclusion:**

Platelet-rich plasma injection can effectively improve the short-term outcomes following arthroscopic repair of full-thickness rotator cuff tears, thus reducing the rate of retears, alleviating pain, and improving patients’ shoulder function. Specifically, the clinical outcomes are better with the use of platelet-rich plasma in single-row fixation than in other fixation techniques. Therefore, platelet-rich plasma injection can be recommended as an adjuvant therapy in single-row repair for improved short-term results.

## Introduction

Rotator cuff tear is a common tendon injury that can result in shoulder pain and limited motor function. Arthroscopic repair is the main treatment for rotator cuff tears, and includes the single-row, double-row, and suture-bridge fixation techniques. Although the surgical technique continues to improve, 8%–94% of patients still suffer from retears[1–5]. To address this, bioremediation techniques, such as platelet-rich plasma (PRP) administration, have been attempted as part of adjuvant therapy in rotator cuff repair.

PRP is a platelet concentrate prepared by centrifugation of autologous whole blood and contains platelet-derived growth factor, transforming growth factor-β, insulin-like growth factor, epidermal growth factor, and vascular endothelial growth factor; as such, PRP promotes cell proliferation and angiogenesis [6]. To date, PRP has been widely used in the beauty industry[7, 8]. Jeong et al.[9] induced photoaging of mouse skin by ultraviolet radiation to produce wrinkles, followed by PRP anti-aging treatment, resulting in a reduction and flattening of wrinkles. However, the effectiveness and safety of PRP in rotator cuff repair are still unclear.

Therefore, the aim of this meta-analysis was to evaluate the clinical effectiveness and safety of platelet-rich plasma administration in arthroscopic repair of rotator cuff injuries and to provide evidence-based medical recommendations for selecting the proper clinical treatment plan. Previous meta-analyses [10–15] evaluated the effects of PRP, platelet-rich fibrin, and plasma rich in growth factors on both full-thickness tears and partial tears; thus, the raw data were heterogeneous. Hence, in the present meta-analysis, we only included studies that used PRP in full-thickness tears; moreover, we reviewed several recently published high-level randomized controlled trials [16–19], which are necessary supplements for current research.

## Methods

### Study selection

Two researchers independently screened the literature, extracted data, and performed crosschecks according to the Preferred Reporting Items for Systematic Reviews and Meta-analyses (PRISMA) strategy[20]. Differences of opinion between the two researchers were resolved by consulting with a third researcher.

### Search strategy

The search strategy was developed based on the guidelines of the Cochrane Collaboration. “Platelet-rich plasma” and “rotator cuff” were used as Mesh words and free words,such as Cuff, Rotator, Infraspinatus, Platelet Rich Plasma, for retrieving the literature from the PubMed, EMBASE, and the Cochrane Library databases by electronic search to September 15,2018(all searches were completed in one day). In addition, the reference lists of all included studies and all literature reviews found in the search results were manually screened for additional articles that met the inclusion criteria.

### Eligibility criteria

Studies that met the following criteria were included in the meta-analysis: (1) presence of full-thickness tears of the rotator cuff in subjects; (2) PRP was administered in the test group, whereas a placebo was administered in the control group or blank control; (3) randomized controlled trials; (4) the follow-up period was at least 12 months; and (5) literature with at least one of the following indicators: retear rate, Constant score, Disabilities of the Arm, Shoulder, and Hand (DASH) score, Visual Analog Scale (VAS) score, University of California at Los Angeles (UCLA) activity score, and/or complications.

The following were excluded from the meta-analysis: (1) animal or cadaveric studies; (2) studies in which it was impossible to extract or convert valid data; and (3) retrospective studies, reviews, and conference papers without full text.

### Data extraction

Two researchers independently extracted data from all available studies according to the predesigned form of data extraction. When the standard deviation was not available, it was converted according to the Cochrane Handbook for Systematic Reviews of Interventions[21]. The risk of bias in randomized controlled trials was assessed using the guidelines stated in the Cochrane Handbook for Systematic Reviews of Interventions[21].

### Outcome measures

It has previously been shown that the effects of PRP administration on rotator cuff tears are greater in the short term than they are in the long term[16].Therefore, the outcome measures in this study were assessed at both the short-term (12 months after surgery) and long-term (≥24 months after surgery) follow-up evaluations. Primary outcomes included the following: (1) retear rate, a common evaluation index for rotator cuff repair that is based on magnetic resonance imaging, computed tomography angiography, and ultrasound inspection, wherein rotator cuff tears are defined as types IV and V of the Sugaya classification[22] or grades III and IV of the Boileau grading system [5]; and (2) Constant score, which includes subjective and objective scores and is the most commonly used indicator for evaluating shoulder function. The following indicators were considered secondary outcome measures: (1) DASH score, an important subjective scoring indicator for evaluating upper limb function, which serves as a supplement to the Constant score; (2) UCLA score, a commonly used shoulder score in North America that includes subjective and objective scores; (3) VAS score, visually quantifies pain conditions in patients; and (4) complications, specifically PRP-related complications, such as hematoma and itchy rash.

### Statistical analysis

Statistical analysis was performed using Review Manager (RevMan) Version 5.3 (Copenhagen: The Nordic Cochrane Centre, The Cochrane Collaboration, 2014). The chi-squared test was used to assess heterogeneity. A random-effects model was used if the value of I^2^ >50%, otherwise, a fixed-effects model was used. Relative risk (RR) was used for dichotomous variables, and standardized mean differences (SMDs) were used for the pooled analysis of continuous variables[23]. Estimates of the 95% confidence interval (CI) and test results of the hypothesis for each variable were listed in a forest plot. For the outcome indicators with significant heterogeneity, a sensitivity analysis was conducted by eliminating the included studies one after the other to ascertain the source of the heterogeneity. Considering that different surgical patterns might cause different therapeutic outcomes, a subgroup analysis was performed according to the surgical pattern[24–28]. Assessment of publication bias using forest plots was intended to be conducted if more than 10 studies were included. The GRADEpro Guideline Development Tool (McMaster University, 2015, developed by Evidence Prime, Inc.) was used to determine the level of evidence.

## Results

### Literature search

**Fig 1:**
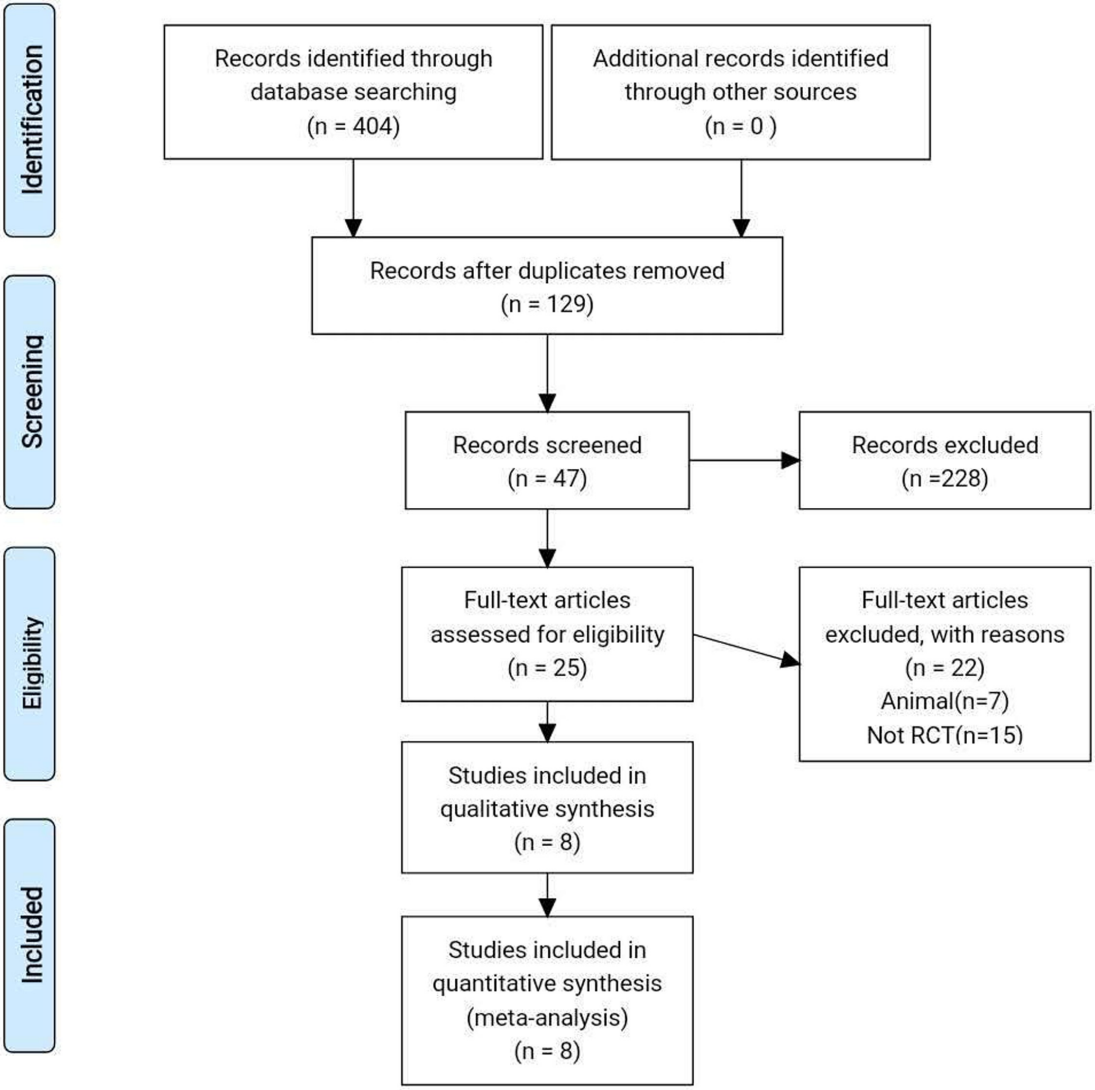
Flowchart of selection of studies.

**Fig 2:**
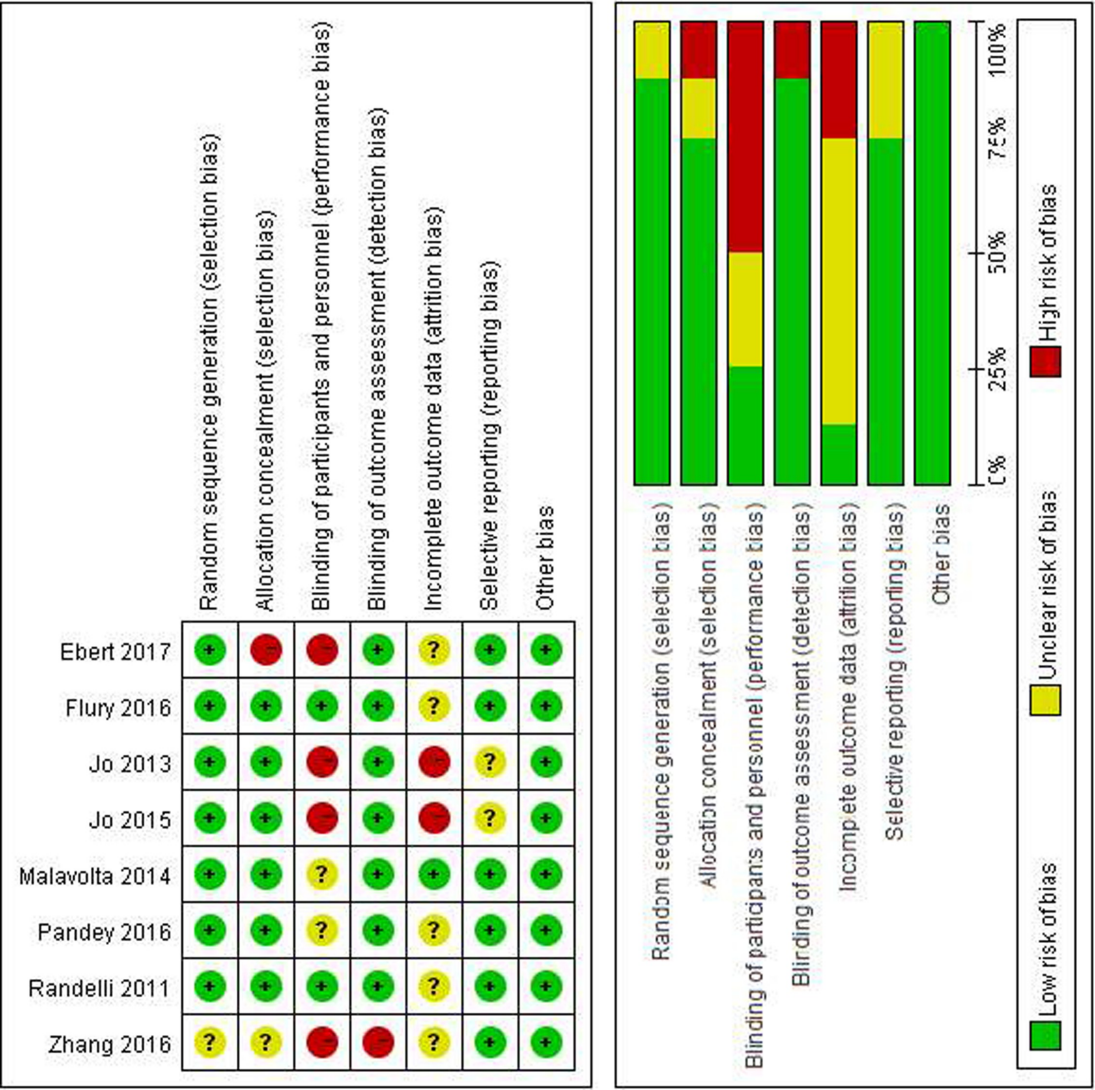
The methodological quality of the included studies.

A total of 404 related studies were consecutively searched and screened. Finally, 8 randomized controlled trials involving 566 patients were included (Fig 1)[16–19, 29–32]. Evaluation of the quality of the reports is shown in Fig 2.

### Study characteristics

There were 283 patients in the PRP treatment group and 283 in the control group; 357 patients underwent single-row fixation and 209 patients underwent double-row fixation. The follow-up period ranged from 12 to 42 months. Basic characteristics of the included studies are shown in Table 1.

**Table 1:** Main characteristics of all eligible studies included in the analysis.

Author	year	Patients(No.)		Mean Age		Male(No.)		Surgery	Imaging	Follow-up (months)
		PRP	Control	PRP	Control	PRP	Control			
Ebert	2017	27	28	59.5	59.7	11	17	double-row	MRI	42
Zhang	2016	30	30	56.9	57.2	15	16	double-row	MRI	12
Flury	2016	60	60	57.8	58.9	18	20	double-row	MRI/US	24
Jo	2015	37	37	61.2	60.9	8	9	double-row	MRI	12
Jo	2013	24	24	64.2	61.9	10	14	double-row	MRI/CTA	12
Pandey	2016	52	50	54.8	54.1	38	36	single-row	US	24
Malavolta	2014	27	27	55.3	54.7	8	9	single-row	MRI	24
Randelli	2011	26	27	61.6	59.5	8	13	single-row	MRI/MRA/US	24

### Clinical outcomes

#### Short-term retear rate

**Fig 3:**
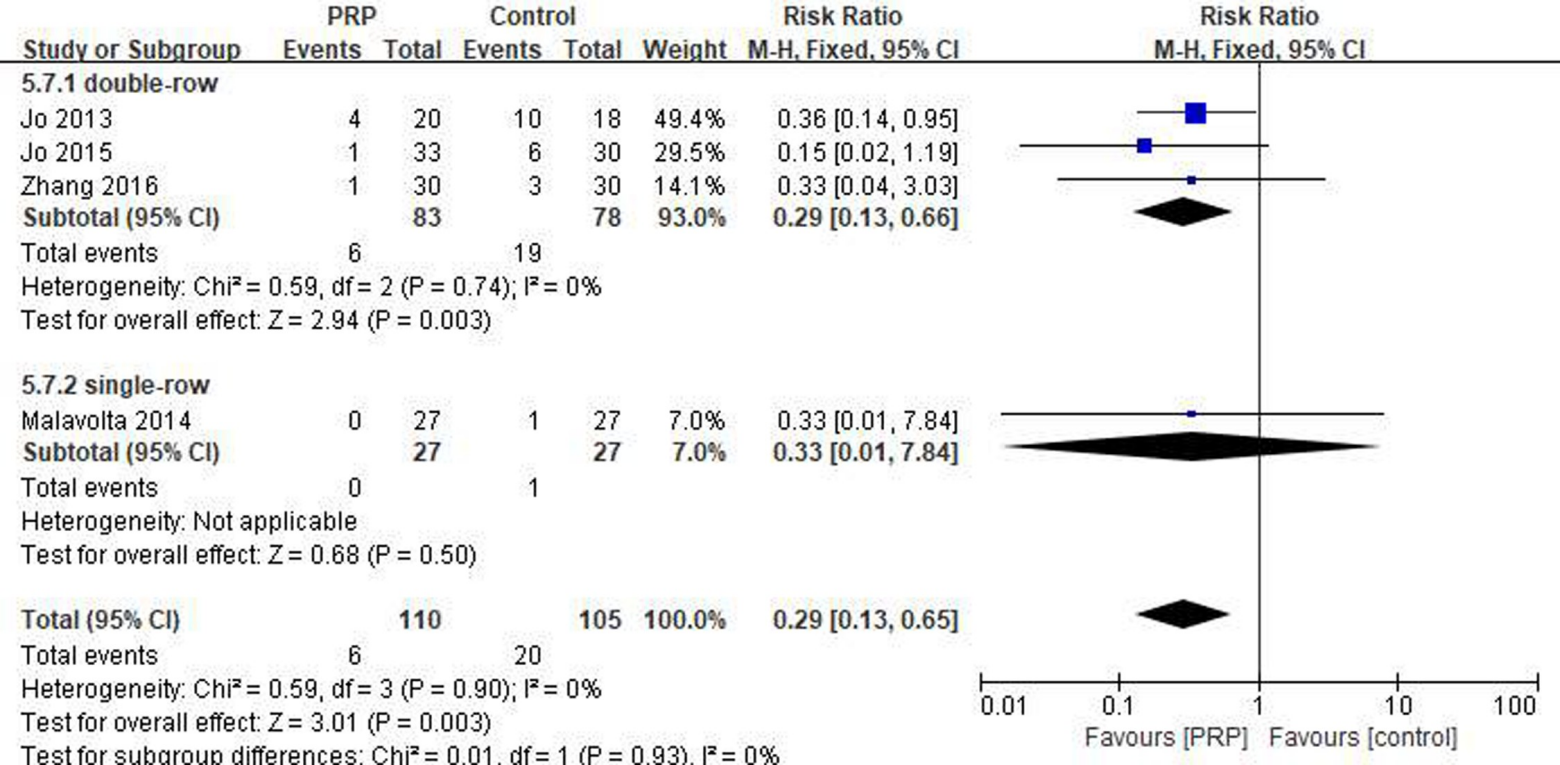
Forest plot for the short-term retear rate.

Four studies[19, 29–31] reported on the short-term retear rate, including 110 patients in the PRP group and 105 in the control group. The homogeneity across the studies was good (I^2^ = 0%, P = .90), and the fixed-effects model was selected. The short-term retear rate was significantly lower in the PRP group than in the control group (RR = 0.29, 95% CI [0.13, 0.65], P = .003) (Fig 3). In the subgroup analysis, a statistical difference in the short-term retear rate between the PRP and control groups was observed for double-row fixation (RR = 0.29, 95% CI [0.13, 0.66], P = .003) but not for single-row fixation (RR = 0.33, 95% CI [0.01, 7.84], P = .5). The GRADEpro assessment showed a moderate level of evidence for the aforementioned results.

#### Long-term retear rate

**Fig 4:**
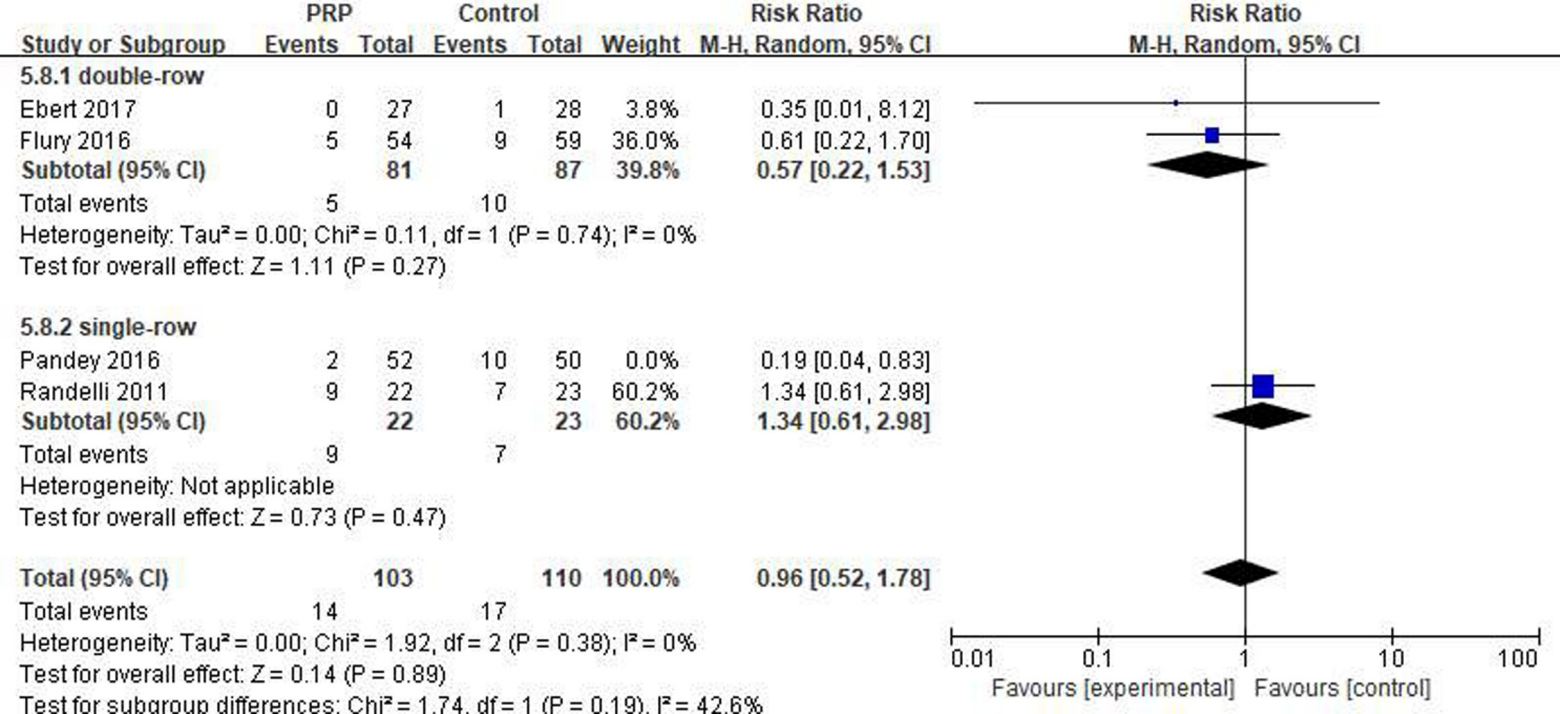
Forest plot for the long-term retear rate.

Four studies[16–18, 32] reported on the long-term retear rates, including 155 patients in the PRP group and 160 in the control group. The I^2^ value was 51%, indicating heterogeneity (P = .90), and thus the study by Pandey et al. [18] was removed, which reduced the value of I^2^ to 0%, indicating good homogeneity (P = .38). No significant difference in the long-term retear rate was identified between the PRP and control groups (RR = 0.96, 95% CI [0.52, 1.78], P = .89). In the subgroup analysis, no statistical differences in the long-term retear rate between the PRP and control groups were observed with respect to single-row fixation (RR = 1.34, 95% CI [0.61, 2.98], P = .47) and double-row fixation (RR = 0.57, 95% CI [0.22, 1.53], P = .27), as shown in Fig 4. The quality of the above evidence was identified as moderate.

#### Short-term Constant score

**Fig 5:**
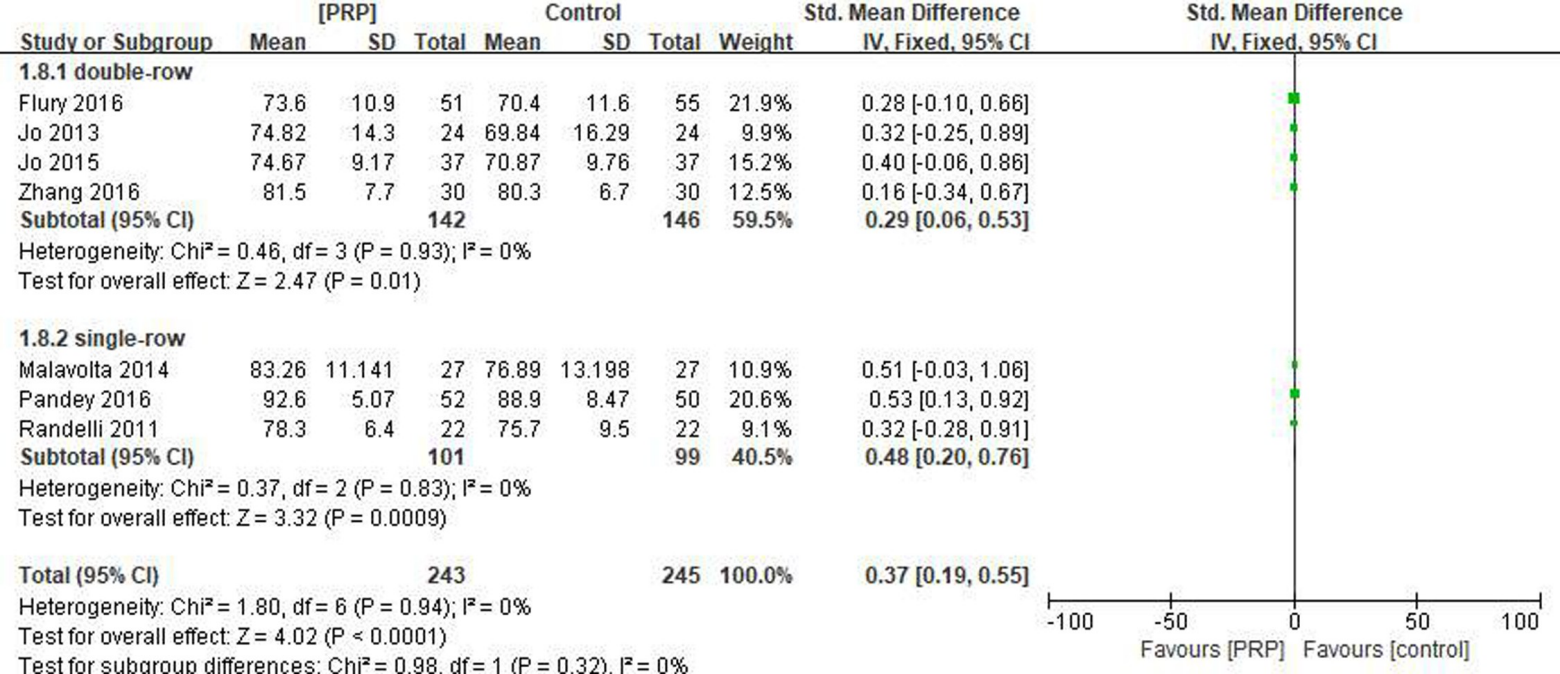
Forest plot for the short-term constant score.

Seven studies[17–19, 29–32] reported on the short-term Constant scores, including 243 patients in the PRP group and 245 patients in the control group. The homogeneity across the studies was good (I^2^ = 0%, P = .94), and the fixed-effects model was selected. The short-term Constant score was higher in the PRP group than in the control group (SMD = 0.37, 95% CI [0.19, 0.55], P < .0001). In the subgroup analysis, the short-term Constant scores were significantly different between the PRP and control groups for single-row fixation (SMD = 0.48, 95% CI [0.20, 0.76], P = .0009) and double-row fixation (SMD = 0.29, 95% CI [0.06, 0.53], P = .01) (Fig 5). The quality of the above evidence was rated as moderate.

#### Long-term Constant score

**Fig 6:**
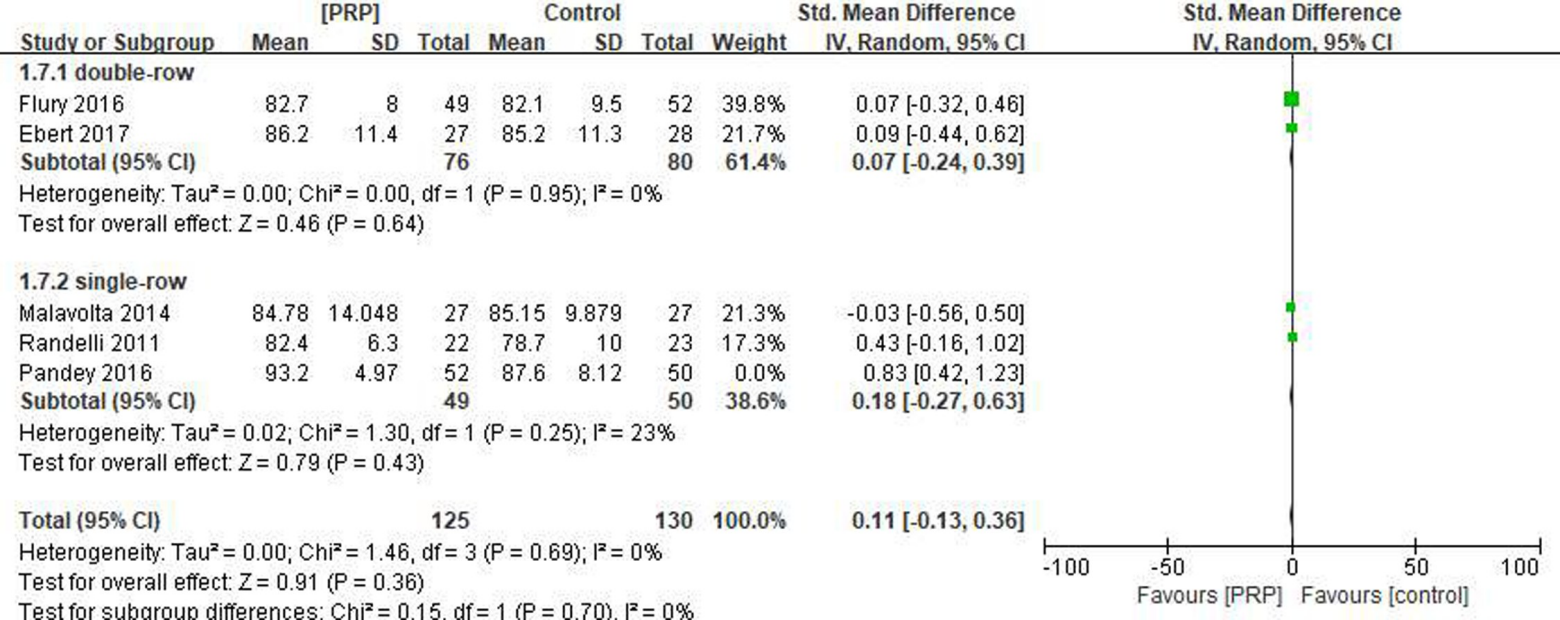
Forest plot for the long-term constant score.

Five studies[16–18, 31, 32] reported on the long-term Constant scores, including 177 patients in the PRP group and 180 patients in the control group. As the value of I^2^ was 61%, indicating heterogeneity (P = .04), the study by Pandey[18] was excluded. In doing so, the value of I^2^ was reduced to 0%, indicating good homogeneity (P = .69). There was no statistical difference in the long-term Constant scores between the PRP and control groups (SMD = 0.11, 95% CI [-0.13, 0.36], P = .36). In the subgroup analysis, no statistical differences in the long-term Constant scores were identified between the PRP and control groups for single-row fixation (SMD = 0.18, 95% CI [-0.27, 0.63], P = .43) and double-row fixation (SMD = 0.07, 95% CI [-0.24, 0.39], P = .64) (Fig 6). The quality of the above evidence was rated as moderate.

#### Short-term DASH score

**Fig 7:**
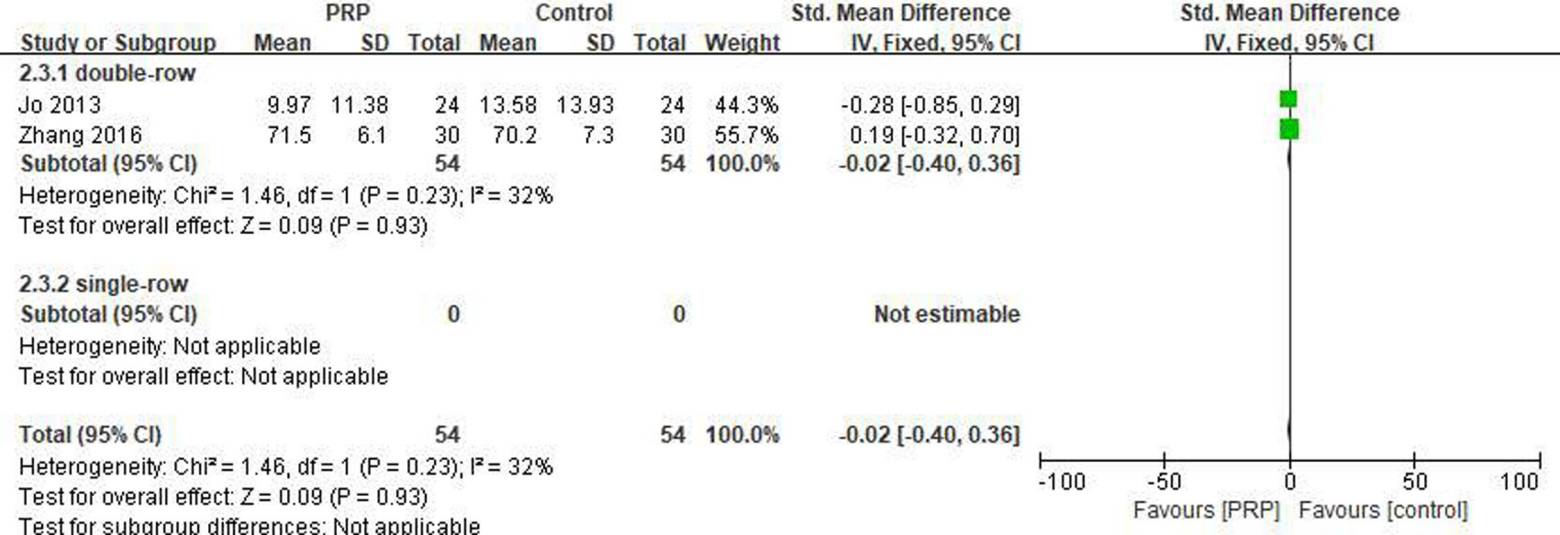
Forest plot for the short-term DASH score.

Two studies[19, 30] reported on the short-term DASH scores, including 54 patients in the PRP group and 54 patients in the control group. The homogeneity between the studies was good (I^2^ = 32%, P = .23), and the fixed-effects model was selected. No statistical differences in the short-term DASH scores were noted between the PRP and control groups (SMD = -0.02, 95% CI [-0.40, 0.36], P = .93) (Fig 7). The quality of the above evidence was rated as moderate.

#### Long-term DASH score

**Fig 8:**
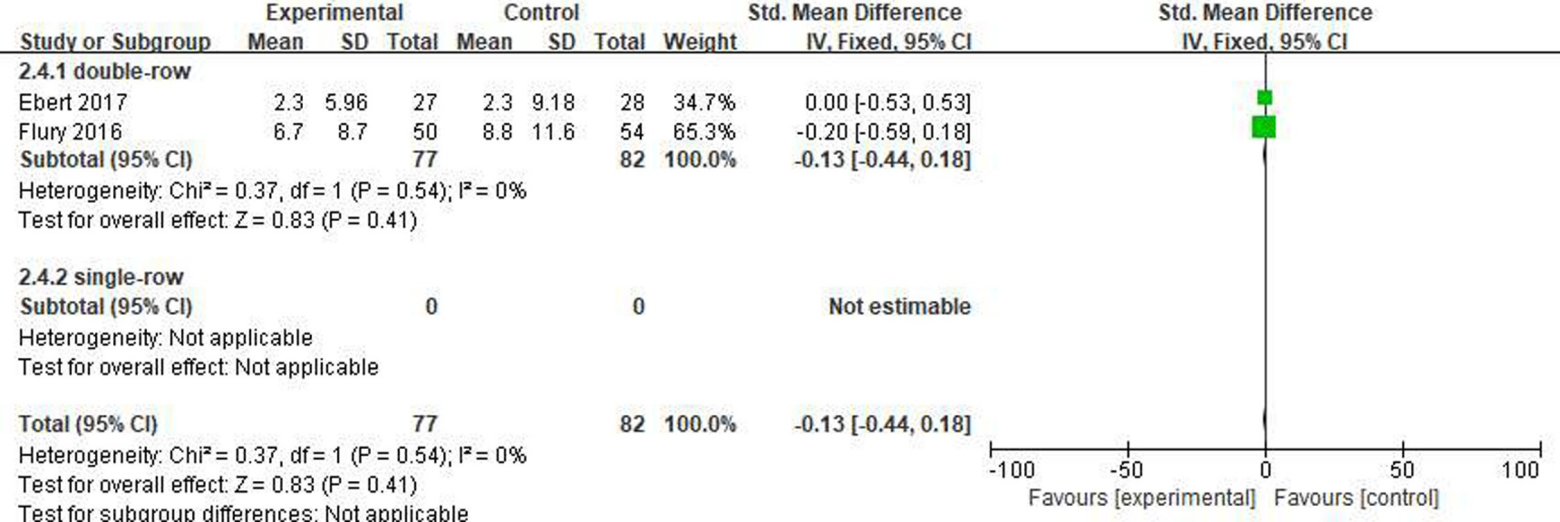
Forest plot for the long-term DASH score.

Two studies[16, 17] reported on the long-term DASH scores, including 77 patients in the PRP group and 82 patients in the control group. The homogeneity between the studies was good (I^2^ = 0%, P = .54), and the fixed-effects model was selected. There were no statistical differences in the DASH scores between the two groups (SMD = -0.13, 95% CI [-0.44, 0.18], P = .41) (Fig 8). The quality of the above evidence was rated as moderate.

#### Short-term UCLA score

**Fig 9:**
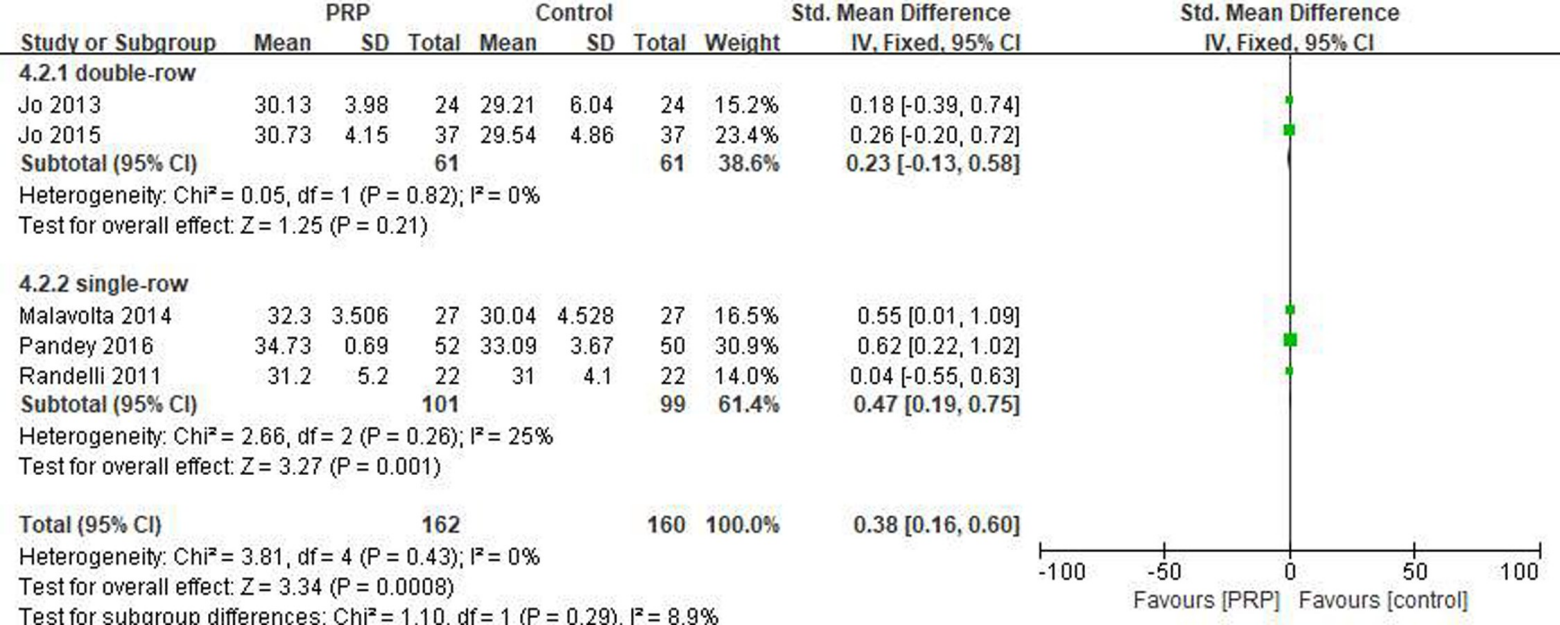
Forest plot for the short-term UCLA score.

Five studies [18, 29–32] reported on the short-term UCLA scores, including 162 patients in the PRP group and 160 patients in the control group. The homogeneity across the studies was good (I^2^ = 0%, P = .43), and the fixed-effects model was selected. The short-term UCLA score was significantly higher in the PRP group than in the control group (SMD = 0.38, 95% CI [0.16, 0.60], P = .0008). In the subgroup analysis, a statistical difference in the short-term UCLA score between the PRP and control groups was observed for single-row fixation (SMD = 0.47, 95% CI [0.19, 0.75], P = .001) but not for double-row fixation (SMD = 0.23, 95% CI [-0.13, 0.58], P = .21) (Fig 9). The quality of the above evidence was rated as moderate.

#### Long-term UCLA score

**Fig 10:**
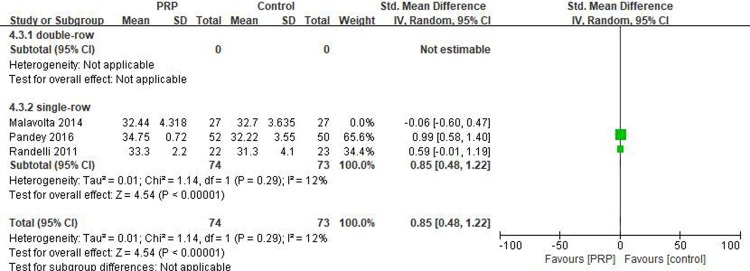
Forest plot for the long-term UCLA score.

Three studies[18, 31, 32] reported on the long-term UCLA scores, including 74 patients in the PRP group and 73 patients in the control group. There was heterogeneity between the studies (I^2^ = 79%, P = .009), and the study by Malavolta et al. [31] was excluded. The value of I^2^ was then reduced to 12%, indicating better homogeneity (I^2^ = 12%, P = .29). The long-term UCLA score was significantly higher in the PRP group than in the control group (SMD = 0.85, 95% CI [0.48, 1.22], P < .00001) (Fig 10). The quality of the above evidence was rated as low.

#### Short-term VAS score

**Fig 11:**
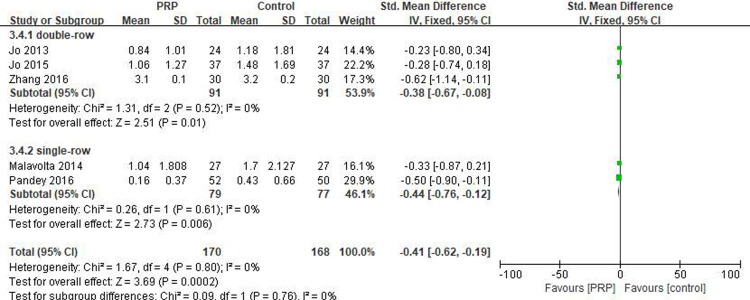
Forest plot for the short-term VAS.

Five studies[18, 19, 29–31] reported on the short-term VAS scores, including 170 patients in the PRP group and 168 patients in the control group. The homogeneity across the studies was good (I^2^ = 0%, P = .80), and the fixed-effects model was selected. The short-term VAS score was significantly lower in the PRP group than in the control group (SMD = -0.41, 95% CI [-0.62, -0.19], P = .0002). In the subgroup analysis, there were statistical differences in the short-term VAS score between the PRP and control groups with respect to single-row fixation (SMD = -0.44, 95% CI [-0.76, -0.12], P = .006) and double-row fixation (SMD = -0.38, 95% CI [-0.67, -0.08], P = .01) (Fig 11). The quality of the above evidence was rated as moderate.

#### Long-term VAS score

**Fig 12:**
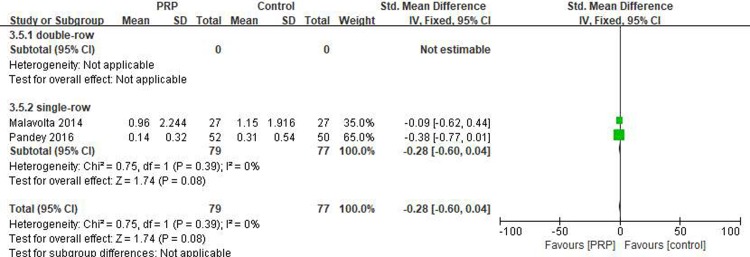
Forest plot for the long-term VAS.

Two studies[18, 31] reported on the long-term VAS scores, including 79 patients in the PRP group and 77 patients in the control group. The homogeneity between the studies was good (I^2^ = 0%, P = .39), and the fixed-effects model was selected. There was no statistical difference in the long-term VAS score between the two groups (SMD = -0.28, 95% CI [-0.60, 0.04], P = .08) (Fig 12). The quality of the above evidence was rated as low.

#### Complications

Only the study by Flury et al. [17] reported complications (Table 2).

**Table 2:** Adverse event reported in the Flury’s study.

Adverse Event (May be related to injection of PRP)	PRP/Control
Infection	1/0
Hematoma (leading to additional treatment)	1/0
Skin problem (exanthematous itchy skin lesion)	1/0
Incidence rate	5.6%/0%

## Discussion

Herein, we performed a meta-analysis to determine the clinical effectiveness and safety of platelet-rich plasma administration in arthroscopic repairs of rotator cuff tears in order to provide evidence-based medical recommendations for selecting an appropriate clinical treatment plan for full-thickness rotator cuff injuries.

Based on the findings of the present study, the short-term retear rate, short-term Constant score, short-term UCLA score, short-term VAS score, and long-term UCLA score were better in the PRP group than they were in the control group, whereas the long-term retear rate, long-term Constant score, long-term DASH score, long-term VAS score, and short-term DASH score were not statistically different between the two groups. Only one of the included studies reported related complications[17]. Except for the long-term UCLA score and long-term VAS score, the level of evidence for all of the indicators was rated as moderate.

The results of the sensitivity analysis indicated that for the long-term retear rate and long-term Constant score, the source of heterogeneity was the study by Pandey et al.[18],and for the long-term UCLA score, the source of heterogeneity was the study by Malavolta et al.[31].

The retear rate is an important outcome measure for the treatment of rotator cuff tears. The natural healing of the tendon involves three stages, namely inflammation, proliferation, and remodeling, and this process is regulated by various growth factors. PRP contains a large number of growth factors, which can promote cell proliferation and differentiation, contribute to wound healing [33–37], and significantly lower the short-term retear rate. In the subgroup analysis, the PRP group had a significant effect with respect to double-row fixation, but there was no statistically significant difference for single-row fixation. Considering that there was only one study addressing the single-row fixation technique [31], its conclusion needs further verification. In the study on long-term retear rates [16–18, 32], the effect of PRP was weakened due to compensation by the patient’s own healing ability; therefore, no statistical difference was observed between the PRP and control groups.

Treatment of rotator cuff injury ultimately aims to restore shoulder function, and this was evaluated in the present study with the Constant score, UCLA score, and DASH score. Based on the aforementioned findings, the Constant score showed the same results, i.e., significant short-term therapeutic efficacy was achieved in patients, while the long-term efficacy showed no significant difference. Both short-term and long-term UCLA scores showed that PRP administration significantly improved the patients’ shoulder function after full-thickness rotator cuff injury, but the quality of evidence for the long-term UCLA scores was rated as low by the GRADEpro software. Therefore, further research on this aspect is required. There was no statistical difference between the short-term and long-term DASH scores, probably because the DASH score is the upper limb function score, which is affected by multiple joints and is not determined only by healing of the rotator cuff. In addition, the original randomized controlled trial design did not involve secondary outcomes; type II errors existed in the statistical design, and thus, a larger sample size is required for verification[16]. There is a ceiling effect in the measurement of patient outcome indicators, resulting in poor sensitivity of the DASH score to the evaluation of shoulder function [16], i.e., the DASH score may be a false negative, needing further verification. The subgroup analyses of the short-term Constant score and UCLA score indicate that PRP plays a role in single-row fixation, whereas only the short-term Constant score was affected in double-row fixation. Therefore, we conservatively infer that PRP can only promote the recovery of short-term shoulder function, especially in single-row fixation.

The evaluation of pain is also a common clinical indicator. Based on the short-term VAS score, we found that PRP can effectively reduce postoperative pain in both single- and double-row fixation; however, details of the underlying mechanism are still unclear. Asfaha et al. [38] speculated that protease-activated receptor-4 in platelets may have an analgesic effect and discovered a potentially endogenous analgesic mechanism associated with protease-activated receptor-4 that reduces hyperalgesia and allodynia in combination with various inflammatory responses. Pandey [18] stated that the analgesic effects of PRP are dose-dependent and that proper PRP can exert good analgesic effects. We speculate that PRP, which contains multiple anti-inflammatory factors [39], can reduce early stage pain that is mainly caused by inflammation and joint stiffness. Such mild pain in the early stage can be managed with functional exercises to prevent severe joint stiffness.

The difference in the efficacy of PRP injections between different surgical methods is due to the high mechanical strength of double-row fixation, which minimizes the formation of the gap and maintains the mechanical stability until healing by increasing the strength of fixation. Hence, the auxiliary effect of PRP injection is not obvious, especially in small rotator cuff tears. However, the mechanical strength of single-row fixation is low and the retear rate is high, and thus the injection of PRP can help reduce the retear rate [40–42].

Except for the study by Flury et al. [17], there are no reports of complications related to PRP injections. As a biological agent derived from autologous blood, PRP has no obvious immunogenicity, and standard PRP injections have good safety and can completely eliminate the side effects caused by mechanical damage.

### Limitations

Our study had a few limitations. First, since a difference exists in the original randomized controlled trial protocol, there are not enough studies addressing different outcome measures, and the level of the quality of evidence is mostly rated as moderate. Therefore, high-quality randomized controlled trials with large sample sizes are warranted in the future. Second, there is no uniform standard for the preparation of PRP or its application, which may have caused some heterogeneity across the included studies.

## Conclusions

In the arthroscopic repair of full-thickness rotator cuff injury, the injection of PRP is a safe and effective adjuvant treatment that can significantly improve early outcomes. We conservatively recommend PRP injection as an adjuvant therapy in patients with early functional needs, especially in single-row fixation. High-quality randomized controlled trials with large sample sizes are warranted in the future to validate these findings.

## Supporting information

S1 Checklist (DOC)
